# Development of a core data set for describing, measuring and reporting the learning curve in studies of novel invasive procedures: study protocol

**DOI:** 10.1136/bmjopen-2024-084252

**Published:** 2024-07-25

**Authors:** Jozel Ramirez, Christin Hoffmann, Neil Corrigan, Matthew Kobetic, Rhiannon Macefield, Daisy Elliott, Jane Blazeby, Shelley Potter, Deborah D Stocken, Kerry Avery, Natalie S Blencowe

**Affiliations:** 1 National Institute for Health and Care Research Bristol Biomedical Research Centre, Bristol Centre for Surgical Research, Bristol Medical School, Population Health Sciences, University of Bristol, Bristol, UK; 2 North Bristol NHS Trust, Bristol, UK; 3 Leeds Institute of Clinical Trials Research, University of Leeds, Leeds, UK; 4 University Hospitals Bristol and Weston NHS Foundation Trust, Bristol, UK

**Keywords:** SURGERY, Adult surgery, Minimally invasive surgery

## Abstract

**Abstract:**

**Introduction:**

The introduction of novel surgical techniques and procedures remains poorly regulated and standardised. Although the learning curve associated with invasive procedures is a critical part of innovation, it is currently inconsistently defined, measured and reported. This study aims to develop a core data set that can be applied in all studies describing or measuring the learning curve in novel invasive procedures.

**Methods:**

A core data set will be developed using methods adapted from the Core Outcome Measures in Effectiveness Trials initiative. The study will involve three phases: (1) Identification of a comprehensive list of data items through (a) an umbrella review of existing systematic reviews on the learning curve in surgery and (b) qualitative interviews with key stakeholders. (2) Key stakeholders (eg, clinical innovators, clinicians, patients, methodologists, statisticians, journal editors and governance representatives) will complete a Delphi survey to score the importance of each data item, generating a shortened list. (3) Consensus meeting(s) with stakeholders to discuss and agree on the final core data set.

**Ethics and dissemination:**

The study is approved by an Institutional Ethics Committee at the University of Bristol (ref: 111362). Participants will complete written informed consent to participate. Dissemination strategies include scientific meeting presentations, peer-reviewed journal publications, patient engagement events, use of social media platforms, workshops and other events.

**STRENGTHS AND LIMITATIONS OF THIS STUDY**

- Established and robust methods have been adapted (from the Core Outcome Measures in Effectiveness Trials initiative) to develop the core data set that will allow a standardised approach in describing, measuring and reporting the learning curve in novel invasive procedures.
- Development of the core data set will involve national, multistakeholder input including, but not limited to patients, clinical innovators, methodologists, statisticians and journal editors.
- Multiple data sources are used beyond traditional systematic reviews to generate a comprehensive list of data items.
- Further work will be required to determine the most appropriate way to disseminate, implement and monitor the use of the core data set when describing the learning curve in novel invasive procedures.

## Introduction

 Innovation has transformed modern-day surgery and improved patient outcomes through the development of techniques such as minimally invasive surgery.^1 2^ The introduction and evaluation of novel techniques and procedures, however, remains poorly regulated and unstandardised, with potentially disastrous consequences (eg, the documented harms associated with metal-on-metal hip implants and vaginal mesh as highlighted in the Cumberlege review).^3^,^5^ What makes a procedure ‘innovative’ or ‘novel’ remains difficult to define; it can be characterised as a new or modified procedure that differs from currently accepted local practice, the outcomes of which have not been fully systematically evaluated and reported in a standardised manner, and which may entail unknown outcomes to the patient.^6 7^

A critical aspect of surgical innovation is the ‘learning curve’, which encompasses improvements in surgeon performance or proficiency with increasing experience, theoretically conferring better patient outcomes over time.^8^ To improve the process of introducing and evaluating novel procedures, the IDEAL (Idea, Development, Exploration, Assessment, Long-term follow-up) collaboration proposed a prospective, stepwise framework for the design and reporting of studies of surgical innovation in a transparent manner.^1 9^ Although the surgical learning curve is acknowledged within stages 2b/3 of the IDEAL framework,^10^ details of how it should be described, measured and reported are not discussed.

Common ways of measuring the learning curve for novel procedures include short-term parameters such as operative time and measures of adverse events such as intraoperative blood loss.^11^,^13^ Although readily available and easily quantifiable, these outcomes may not necessarily reflect the best or most comprehensive way to assess improvements in surgical performance, or be of greatest relevance to clinicians, patients and other stakeholders or to decision-making regarding further surgeon training or skill acquisition. A lack of standardisation when describing and measuring the learning curve of novel procedures may also complicate or hinder progression to the next stage of evaluation.^10^ Work is, therefore, needed to improve consistency in the selection and reporting of ‘learning curve outcomes’ in studies of surgical innovation.

One possible solution to improve standardisation of describing and measuring the surgical learning curve is to develop a core data set—an agreed minimum set of data items that should be measured and reported in all studies describing and assessing the learning curve.^14^ There is increasing support for the development and use of core sets to ensure that important outcomes are measured consistently across a range of disciplines, including surgery.^15^,^17^ For example, a generic core outcome set for surgical innovation has recently been developed (the COHESIVE core outcome set) to specify *what* outcomes are important.^18^ Operators’/surgeons’ experience of performing an innovative procedure was identified as one of eight core outcome domains, and a recommended next step is to identify *how* it should be measured. Detailed guidance for describing, measuring and reporting the learning curve in surgical innovation can complement this work to ultimately improve standardisation and promote robust evaluation in this area. This study aims to achieve consensus on the minimum set of data items to be reported in all studies describing or measuring the learning curve for novel invasive procedures.

## Methods

### Overview

**Figure 1:**
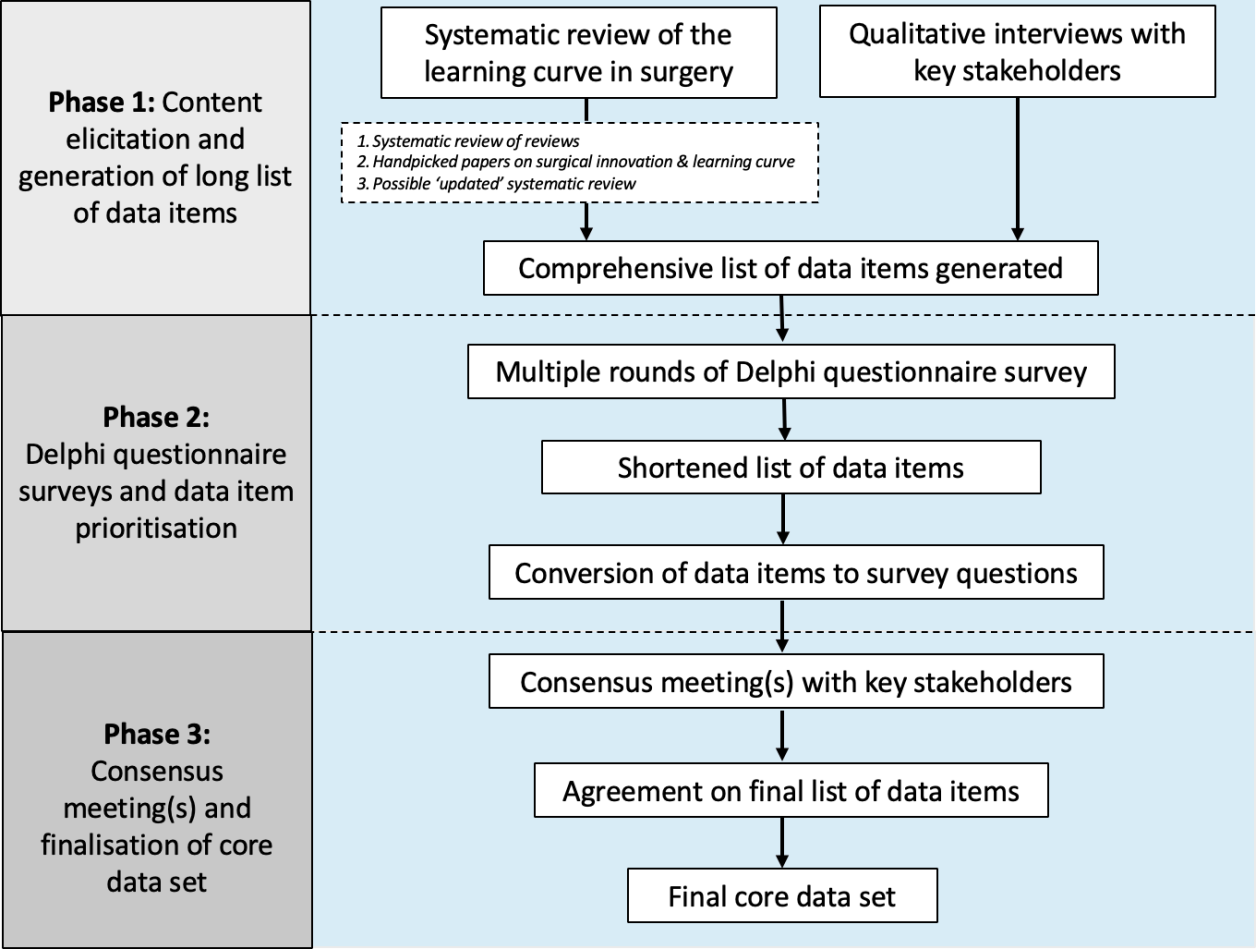
Summary of the three phases involved in the development of the core data set.

Methods for developing the core data set have been adapted from the Core Outcome Measures in Effectiveness Trials (COMET) initiative.^14^ The study has been registered in the COMET database (https://www.comet-initiative.org/Studies/Details/2861). It will involve three phases, as summarised in figure 1. The study was initiated in January 2023, with a provisional completion date of January 2026.

### Phase 1: identification of a ‘long list’ of data items and semistructured interviews

Phase 1 will generate a ‘long list’ of items by conducting (a) an umbrella review of existing systematic reviews describing/measuring/reporting the surgical learning curve, and (b) qualitative interviews with key stakeholders. This will inform the development of a Delphi questionnaire (phase 2).

#### Umbrella review

An umbrella review (or ‘review of reviews’) will be conducted to identify all relevant studies involving the learning curve in surgery.^19^ We hypothesise this will be the most efficient method to map out a broad and complex topic as a scoping exercise to identify existing literature identified a large number of relevant systematic reviews.

##### Aim

The aim of this umbrella review is to examine reviews of studies that focus on the learning curve in surgery. The product of this review will be used to generate a long list of items that informs other parts of this phase of the study.

##### Methods

###### Search strategy

Published review articles will be identified by systematic searches in Ovid MEDLINE/Embase, Cochrane Library and Epistemonikos^20^ electronic databases. Searches will be limited to the past 10 years to ensure only contemporary studies are included. Searches will consist of subject headings and text words combining terms related to surgery, invasive procedures, the learning curve and systematic reviews. Existing searches related to ‘invasive procedures’ and ‘systematic reviews’ will be used and adapted as necessary.^11 13 21^

Review articles will be supplemented by further data sources to ensure contemporaneous literature is included. This will include, for example, identification of additional relevant published reviews of surgical innovation studies through expert knowledge.^16^ Supplemental searches for studies involving the learning curve in surgery will be conducted if there are deficiencies in the existing reviews identified (eg, outdated papers and lack of relevance).

###### Selection and eligibility of papers

Scoping and systematic reviews that focus on the learning curve in surgery/minimally invasive procedures available in English will be eligible. Invasive procedures will be defined as those where access is gained via an incision, natural orifice or percutaneous puncture, or involving devices used inside the body.^22^ This will include:

- Review articles that aim to investigate the surgical learning curve for an invasive surgical procedure/device (this can include, eg, a specific surgical technique, procedure or approach).
- Review articles that describe/assess/report literature on the learning curve for an invasive surgical procedure/device (this may include, eg, reviews that summarise results of the surgical learning curve but may not state this as the primary aim of the review).

Excluded will be reviews that assess surgical skills/competency at a specific point in time. Abstracts and conference reports will be excluded due to difficulties in evaluating incomplete information.

A customised inclusion/exclusion form will be used to screen for eligible articles. Screening will be done through a two-stage process (titles/abstracts/keyword screening followed by full-text screening). All titles and abstracts will be screened independently by two authors. All full-texts will be independently assessed further for eligibility by two authors. Any conflicts not resolved by discussion will be referred to the study team for a final decision.

###### Data extraction

A customised electronic form will be used to collect relevant data. Every study included within each systematic review will be accessed to ensure all relevant items are collected. General study characteristics and any method or metric explicitly used to describe or measure the learning curve will be extracted verbatim. This will include quantitative and qualitative data, including all parameters (eg, preoperative, perioperative and postoperative, patient-related, surgeon-related or healthcare system-related). Data from eligible full-texts will be extracted, of which a minimum of 10% will be independently extracted by two authors to check for concordance.

###### Quality appraisal

AMSTAR-2 (A MeaSurement Tool to Assess systematic Reviews) will be used to assess the methodological quality of systematic reviews included within the umbrella review. AMSTAR-2 is a widely cited tool specifically designed to critically appraise systematic reviews of randomised and non-randomised studies.^23^

###### Data analysis

A comprehensive ‘long list’ of unique items will be generated, which will inform the topic guide for the qualitative interviews (see below). Verbatim data will be grouped into domains and duplicates will be removed. Where appropriate, a narrative synthesis of the data will be completed, and descriptive statistics will be used to present findings.

### B) Qualitative interviews with key stakeholders

#### Sampling and recruitment

Identification of any potential further data items will be explored through semistructured interviews with key stakeholders with knowledge/experience of the learning curve in surgery/invasive procedures. Stakeholders will include, but not limited to surgical innovators/adopters, clinicians, methodologists, statisticians, industry partners, governance representatives and patients. They will be identified through professional organisations/meetings, and existing collaborations (eg, Bristol Biomedical Research Centre). Individuals will be invited to participate through communication from their organisation or directly from our research group. Patient participants will be included in all phases and will be recruited using various strategies including personal networks, contact lists held by our research team, online platforms and an existing patient and public involvement and engagement (PPIE) strategy group. Eligible patient participants will be individuals aged 18 years or over who have had experience with surgery. We will use purposive sampling to ensure a diverse range of stakeholders is included, according to age, gender and race and clinical discipline/level of experience where appropriate.

#### Data collection

One-to-one semistructured interviews will be conducted either face-to-face or via video conferencing software/telephone call by members of the research team. All interviews will take place at the participants’ convenience. Interview schedules will be informed by the literature review (see above) and protocol, and expert opinion will be tested and modified to accommodate topics of interest emerging as data collection progresses. Questions will focus on detecting unique concepts not identified in the literature review by exploring stakeholders’ perspectives on the learning curve and the range of data items that could be used to describe, measure or account for it, including clinician and patient-centred outcomes.

Interviews will be audio-recorded and transcribed verbatim. NVivo 12 (QSR International, Melbourne, Australia), Microsoft Excel and Word will be used to aid in the storage and analyses of all types of data. Transcripts of interviews will be coded line-by-line by ascribing keywords or phrases that capture the meaning of the text.^24^ A subset of transcripts will be double-coded independently by a second researcher trained and experienced in qualitative research.

### C) Generating a comprehensive list of items

Following the literature reviews and qualitative interviews, coding results will be reconciled to generate a long list of data items. Duplicates will be removed, and each unique item will be independently mapped and categorised into broader domains by at least two researchers. A domain will be defined as a group of items that are broadly within the same theme. This process will involve multidisciplinary team discussions and iterative refinements of the list and domains until agreement has been reached.

The final list of domains and items will be taken forward to phase 2.

### Phase 2: Delphi questionnaire surveys

The objective of phase 2 is to employ a consensus method involving a sequential, multiround Delphi survey to reduce the long list of items generated in phase 1. The shortened list of items will be carried on to consensus stakeholder meetings in phase 3.

#### Delphi questionnaire development

The long list of domains and data items will be operationalised into a Delphi questionnaire, with each item forming an individual question and domains forming section headings. Items will be written in plain English with clinical terminology included in parentheses. A draft Delphi questionnaire will be piloted using ‘think aloud’ techniques by relevant stakeholders, including surgeons, patients and methodologists examining face validity, comprehension and acceptability.^25^

#### Delphi process

The Delphi process will consist of sequential rounds of questionnaires with the same group of participants, hosted by a secure electronic data capture software (eg, REDCap).^26^ This will be used to prioritise a list of items to be considered in consensus meetings by key stakeholder groups of professionals and patients. The Delphi survey process avoids any effect of dominant individuals by allowing a representative sample of stakeholders to participate anonymously. It is anticipated that at least two Delphi rounds will be conducted, with additional rounds to be considered if appropriate. In each questionnaire, participants will be asked to rate the importance of each item from 1 (not important) to 9 (critically important).^14^

Delphi participants will include, but not be limited to clinicians, clinical innovators/adopters, methodologists, statisticians, journal editors, industry partners, governance representatives and patients. Recruitment will use relevant sources including, but not limited to professional organisations, surgical associations, personal networks, social media, charities and patient support networks. There is no recommended minimum sample size for Delphi surveys. Based on previous similar research, we aim to recruit approximately 200 participants across professional and patient stakeholder groups through purposive sampling.^15^ Participants will have a range of experience, roles, geographical location, age, race and gender to ensure balanced representation. Participants will receive questionnaires electronically and will receive one follow-up reminder if necessary.

#### Statistical analysis and consensus definition

Analysis will be undertaken using Stata (V.15 or later). Following each Delphi round, the average score (eg, median) of data item responses will be calculated and presented as feedback in the subsequent round. In case of merged items, participants’ scores will be calculated as the mean of the individual items’ scores.

Standard guidance to define consensus will be adhered to and adapted if necessary from the COMET initiative and previous studies.^14 15 17^ Items scored 7–9 (critically important) by ≥70% and 1–3 (not important) by <15% of participants will be considered ‘consensus in’. Items scored 7–9 by <50% of participants will be considered ‘consensus out’. If neither criteria are met, the data item can be considered as ‘no consensus’. All items will be retained between rounds 1 and 2 to allow the participants to re-score and consider overall feedback from round 1. Items that are likely to be discussed in consensus meetings (phase 3) will be those that have been categorised as ‘consensus in’ or where consensus is uncertain (‘no consensus’).

### Phase 3: consensus meetings

The final step (phase 3) will use stakeholder consensus meeting(s) to agree on a final core data set. A representative sample of stakeholders will be invited to consensus meeting(s) where the results of the Delphi survey will be summarised. This will include, but not limited to surgical innovators/adopters, clinicians, methodologists, statisticians, industry partners, governance representatives and patients. A purposive sample of 20–25 participants will be invited, ensuring that a diverse group with a range of experience and backgrounds will be included. Participants will be recruited through personal networks and from a list of Delphi survey participants in phase 2. It is anticipated that at least one consensus meeting will be held, and further meetings will be considered if deemed necessary. Meeting(s) will be held face-to-face or virtually, depending on logistics and participant preferences.

During the meeting(s), a nominal group technique will be used to gain consensus on the final list for the core data set.^14^ Following a detailed discussion of retained items from the Delphi surveys, participants will be asked to vote on the list of items carried forward using an anonymised system using simple voting options, for example, ‘in’, ‘out’ or ‘unsure’. An independent chairperson will be present to facilitate the meeting. Voting results will be fed back to participants in real time, and conflicts or polarising responses will be discussed further. Cut-off criteria for voting items ‘in’ or ‘out’ will be defined a priori.

### Patient and public involvement and engagement

Patients and public contributors will be involved in all phases of the research. A patient advisory group established within the Bristol Centre for Surgical Research and Bristol National Institute for Health and Care Research Biomedical Research Centre was consulted about the role of patient participation and supported its inclusion. Patient involvement in the study will include, but not be limited to, helping to inform the topic guide, interpretation of interview findings and designing of the Delphi survey.

### Ethics and dissemination

An Institutional Ethics Committee at the University of Bristol has approved the methods of this study (ref: 111362). Participants will complete written informed consent to participate. Dissemination strategies include scientific meeting presentations, peer-reviewed journal publications, patient engagement events, use of social media platforms, workshops and other events.

### Data statement

For research activities where no empirical data will be collected, all relevant data will be submitted in a supplementary file with future publication (related to protocol/research activity). Where empirical data will be collected, these will be anonymised and stored under controlled access on the research data storage facility in line with the University of Bristol data protection and security policies. Applications to access data can be made by emailing rdsf-help@bristol.ac.uk. All applications are subject to committee review.
