# Supplementary material for: Development of a core data set for describing, measuring and reporting the learning curve in studies of novel invasive procedures: study protocol
Source: BMJ Open. 2024 Jul 25;14(7):e084252. doi: 10.1136/bmjopen-2024-084252 (PMC11284869; doi:10.1136/bmjopen-2024-084252)
Supplement: Supplementary file 1 [file bmjopen-2024-084252.reviewer_comments.pdf]

PEER REVIEW HISTORY

BMJ Open publishes all reviews undertaken for accepted manuscripts. Reviewers are asked to complete a checklist review form (**http://bmjopen.bmj.com/site/about/resources/checklist.pdf**) and are provided with free text boxes to elaborate on their assessment. These free text comments are reproduced below.

ARTICLE DETAILS

**TITLE (PROVISIONAL)**	Development of a core data set for describing, measuring and reporting the learning curve in studies of novel invasive procedures: study protocol
**AUTHORS**	Ramirez, Jozel; Hoffmann, Christin; Corrigan, Neil; Kobetic, Matthew; Macefield, Rhiannon; Elliott, Daisy; Blazeby, Jane; Potter, Shelley; Stocken, Deborah; Avery, Kerry; Blencowe, Natalie

VERSION 1 – REVIEW

**REVIEWER**	Rosanne Kho<br>The University of Arizona College of Medicine Phoenix, Ob Gyn
**REVIEW RETURNED**	04-Feb-2024

**GENERAL COMMENTS**	Overall: I commend the study investigators for taking on this important (and enormous) task! Overall, study plan is sound. I have a few specific comments below regarding methodology proposed:<br><br>1. It would be helpful to know early on in the manuscripts which surgeries you intend to include, otherwise, including ALL surgeries that require an incision would be quite broad and may pose a significant challenge at time of data synthesis. As currently defined, in lines 36-40, I foresee that even cardiac, orthopedic, neurosurgical, radiology procedures could be included in this study. The procedures in these fields are quite different from procedures that focus on one anatomic cavity - such as abdomen/pelvis, chest/thoracic, cranium, etc - and their implications to surgical training would also vary greatly. Please clarify.<br>2. Define how many and from which sectors the invited 'stakeholders" participating in the Modified Delphi process. How would you ensure balance of representation to avoid bias?<br>3. Why are the authors opting to omit an assessment of the quality of the studies? Would it not be more efficient and effective to utilize the data (outcome data points) only from the best quality studies? Kindly clarify.

**REVIEWER**	Pooja Prasad<br>Royal Victoria Infirmary
**REVIEW RETURNED**	17-Feb-2024

**GENERAL COMMENTS**	This article is both relevant and timely, as innovative surgical procedures are being increasingly implemented. As a study protocol, steps involved in the development of a core data set are clearly described. Perhaps the author may wish to consider what they define as 'novel surgical procedures' (robotic/laparoscopic/any other modality) and specify this in any revised manuscript. This is to

	account for the great deal of heterogeneity in what is considered 'novel' and is variable across the breadth of surgical specialties.<br><br>Line 58-59 appears incomplete<br><br>Overall, this is a well referenced and succinct protocol paper.

VERSION 1 – AUTHOR RESPONSE

Reviewer 1 comments

Overall: I commend the study investigators for taking on this important (and enormous) task! Overall, study plan is sound.

Reply: We thank the reviewer for their support.

1. It would be helpful to know early on in the manuscripts which surgeries you intend to include, otherwise, including ALL surgeries that require an incision would be quite broad and may pose a significant challenge at time of data synthesis. As currently defined, in lines 36-40, I foresee that even cardiac, orthopedic, neurosurgical, radiology procedures could be included in this study. The procedures in these fields are quite different from procedures that focus on one anatomic cavity - such as abdomen/pelvis, chest/thoracic, cranium, etc - and their implications to surgical training would also vary greatly. Please clarify.

Reply: We thank the reviewer for raising this important point. The aim of our study is to achieve consensus on a minimum set of data items to be reported in all studies describing or measuring the learning curve for novel invasive procedures. We therefore see great value in looking at a wide range of surgical specialties in order to obtain a comprehensive list of possible data items. Although we envisage that there will be additional data items depending on the specific procedure/technique, our main focus is to generate of a minimum set of data items which will be applicable to all novel invasive procedures. Since the submission of this manuscript, we have undertaken searches as part of the umbrella review (Phase 1). Eighty-nine reviews have been identified as eligible for inclusion, which appears to be manageable and in line with other similar reviews.

2. Define how many and from which sectors the invited 'stakeholders" participating in the Modified Delphi process. How would you ensure balance of representation to avoid bias?

Reply: We thank the reviewer for this comment. We aim for maximum variation within our stakeholder group in terms of experience, roles, geographical location, age, race and gender. We will include approximately 200 participants in the Delphi process. Although we are not able to anticipate how many from each stakeholder group will be included, we will aim to achieve a balanced spread across the participants.

Revisions: Amendments have been made for the paragraph below within the 'Delphi process' section on page 11:
"Delphi participants will include, but not be limited to clinicians, clinical innovators/adopters, methodologists, statisticians, journal editors, industry partners, governance representatives and patients. Recruitment will use relevant sources including, but not limited to professional organisations, surgical associations, personal networks, social media, charities, and patient support networks. There is no recommended minimum sample size for Delphi surveys. Based on previous similar research, we aim to recruit approximately 200 participants across professional and patient stakeholder groups through purposive sampling (15). Participants will have a range of experience, roles, geographical location, age, race, and gender to ensure balanced representation. Participants will receive questionnaires electronically and will receive one follow-up reminder if necessary."

3. Why are the authors opting to omit an assessment of the quality of the studies? Would it not be more efficient and effective to utilize the data (outcome data points) only from the best quality studies? Kindly clarify.

Reply: We thank the reviewer for raising this point and agree that it would be useful to describe the quality of included reviews. We will therefore use AMSTAR-2 to appraise the quality of conduct of included papers. AMSTAR (A MeaSurement Tool to Assess systematic Reviews) is a widely cited tool specifically designed to critically appraise systematic reviews of randomised controlled trials. This has been further developed (AMSTAR-2) to enable appraisal of systematic reviews containing non-randomised studies(1).
Of note, we aim to generate a comprehensive list of all data items to inform our 'long list' and we therefore do not intend to exclude studies on the basis of quality, as this may lead to omission of items of potential importance.

Revisions: The 'quality appraisal' section on page 8 has now been changed and reads:
"The main intention of the review is to generate a comprehensive list of data items used to describe, measure and/or report the learning curve in surgery, and therefore a critique of the overall methodological quality of studies will not be undertaken.
AMSTAR-2 will be used to assess the methodological quality of systematic reviews included within the umbrella review. AMSTAR-2 (A MeaSurement Tool to Assess systematic Reviews) is a widely cited tool specifically designed to critically appraise systematic reviews of randomised and non-randomised studies(23)."

Reviewer 2 comments

1. This article is both relevant and timely, as innovative surgical procedures are being increasingly implemented. As a study protocol, steps involved in the development of a core data set are clearly described. Perhaps the author may wish to consider what they define as 'novel surgical procedures' (robotic/laparoscopic/any other modality) and specify this in any revised manuscript. This is to account for the great deal of heterogeneity in what is considered 'novel' and is variable across the breadth of surgical specialties.

Reply: We thank the reviewer for this comment. We acknowledge that, currently, what makes a surgical procedure 'novel' or 'innovative' remains difficult to define in a standardised way. However, there is ongoing progress in this field and previously published work has provided a description of what an innovative invasive/surgical procedure is(2). This is now included within the manuscript.

Revisions: The following sentence has been added to the first paragraph of the 'introduction' section (page 5):
"Innovation has transformed modern-day surgery and improved patient outcomes through the development of techniques such as minimally invasive surgery (1, 2). The introduction and evaluation of novel techniques and procedures, however, remains poorly regulated and unstandardised, with potentially disastrous consequences (e.g. the documented harms associated with metal-on-metal hip implants and vaginal mesh as highlighted in the Cumberlege review)(3-5). What makes a procedure 'innovative' or 'novel' remains difficult to define; it can be characterised as a new or modified procedure that differs from currently accepted local practice, the outcomes of which have not been fully systematically evaluated and reported in a standardised manner, and which may entail unknown outcomes to the patient(6, 7)."

2. Line 58-59 appears incomplete.

Reply: We thank the reviewer for noticing this error. We believe this is a formatting error when converting the figure to PDF, whereby part of Figure 1 has obscured the main text.

Revisions: The complete sentence, found within the aim of the umbrella review (page 7), now reads: “The aim of this umbrella review is to examine reviews of studies that focus on the learning curve in surgery. The product of this review will be used to generate a long list of items that informs other parts of this phase of the study.”

3. Overall, this is a well referenced and succinct protocol paper.

Reply: We thank the reviewer for their support.
